# Age and depressive symptoms change predict the 4‐year self‐concept trajectory for youth after anxiety treatment

**DOI:** 10.1002/jclp.23427

**Published:** 2022-08-16

**Authors:** Krister W. Fjermestad, Katarina Bellika, Caroline Matre, Wendy K. Silverman, Gro Janne Wergeland

**Affiliations:** ^1^ Department of Psychology University of Oslo Oslo Norway; ^2^ Yale Child Study Centre Program for Youth Anxiety Disorders Yale University New Haven Connecticut USA; ^3^ Åsane Child and Adolescent Mental Health Clinic Bergen Norway; ^4^ Haukeland University Hospital Bergen Norway

**Keywords:** anxiety, children, cognitive behavioral therapy, depression, self‐concept

## Abstract

**Objectives:**

To investigate the self‐concept trajectory from before to 4 years after cognitive behavioral therapy (CBT) for youth with anxiety disorders, including predictors.

**Methods:**

Youth with anxiety diagnoses (*N* = 179; *M* = 11.5 years, SD = 2.1; 53.6% girls; 46.4% boys) received CBT in community clinics. Self‐concept, anxiety/depression symptoms, and diagnostic status were assessed at pre‐, post, 1‐year, and 4‐year posttreatment.

**Results:**

Growth curve analyses showed that the self‐concept improved significantly over time (*d* = 0.07 to 0.34). Higher age and a decrease in the depressive symptom trajectory predicted increased self‐concept trajectory from baseline to 4 years posttreatment. Not dropping out of treatment also contributed positively to the self‐concept trajectory, but not above and beyond decreased depressive symptoms. The correlation between self‐concept and depressive symptoms was *r* = 0.60, indicating these are related but distinct.

**Conclusion:**

Self‐concept can improve after CBT, also long‐term. This change appears to primarily be associated with decreased depressive symptoms over time.

Up to 28% of youth develop anxiety disorders, and up to 60% of these youth have a comorbid mood disorder (Seligman & Ollendick, 2011). The core features of anxiety disorders include unhelpful behaviors (e.g., avoiding feared situations), cognitions (e.g., thinking “*I cannot cope*”), and negative emotional physiological reactions (e.g., breathing difficulties), which reciprocally reinforce each other (Lebowitz et al., 2018). Over time, such anxiety features are likely to influence how youth feel about themselves. For example, anxiety‐related cognitions and emotions may lead to more generalized negative thoughts about the self, such as “*I am not a normal person*” (Bleidorn et al., 2016). Poor self‐concept, defined herein as the sum of an individual's system of cognitive and affective beliefs about themselves Busch et al. (2021), is also a core depression symptom (World Health Organization, 2016).

Poor self‐concept has been empirically linked with anxiety and depression symptoms, both as a risk factor (Henriksen et al., 2017), and as a consequence (Stadelmann et al., 2017). The self‐concept has also been associated with broader domains such as quality of life, personality, attitudes, and cognitive style (e.g., Seligman & Ollendick, 2011; Vaquero‐Solís et al., 2021). Therefore, it is important to consider the self‐concept as an outcome in anxiety disorders.

Cognitive behavioral treatment (CBT) is the recommended treatment for youth anxiety disorders (James et al., 2020). Few studies have examined potential CBT effects for the self‐concept, although CBT has been proposed to affect the self‐concept by researchers (e.g., Shirk et al., 2003; Taylor & Montgomery, 2007). Two prevention studies using the FRIENDS for life CBT program showed positive self‐concept changes. A randomized controlled trial with 320 children and adolescents aged 6–19 years compared FRIENDS to a waitlist control (Barrett et al., 2003). Self‐concept improvement was significantly larger from pre to post in the active condition. There was no significant change over time in self‐concept for the waitlist condition (Barrett et al., 2003). The other study was an open trial with 109 children aged 9 and 10 years, which showed significant pre to post self‐concept improvement (Stallard et al., 2008). However, the effect of CBT on the self‐concept in samples with clinical levels of anxiety is still largely unknown. Given that the self‐concept may be more negatively affected for youth with more severe symptoms, it is important to examine self‐concept trajectories in clinical settings, which may differ from prevention settings (Lebowitz et al., 2018).

The aims of the current study are to examine self‐concept over time in a sample of youth who received manual‐based CBT for anxiety disorders, and predictors of the self‐concept trajectory. The data are from a randomized controlled effectiveness trial comparing individual CBT (ICBT) to group CBT (GCBT) delivered in community clinics, with clients who were referred through regular clinical routines (Wergeland et al., 2014). The research question is: How does the self‐concept develop from pretreatment to 4‐year follow‐up for youth with anxiety disorders, and what predicts the self‐concept trajectory? We include youth characteristics, diagnostic and symptom profile variables, and treatment factors as predictors of the self‐concept trajectory.

In terms of youth characteristics, we include youth sex and age. Sex differences in the self‐concept are unresolved. Most studies show that girls are more at risk for low self‐concept and anxiety than boys (e.g., Bleidorn et al., 2016), but the picture is complex. According to a review (Robins & Trzesniewski, 2005), boys and girls have similarly high self‐concept in childhood, but the self‐concept decline in adolescence is steeper for girls. Stadelmann et al. (2017) found that when mental health disorders were present, both sexes were affected in most self‐concept domains. We include youth age because the normative developmental trajectory of the self‐concept is unresolved (Orth et al., 2018).

In terms of diagnostic and symptom profile variables, we include social phobia as a primary diagnosis because several studies have found reduced self‐concept for social phobia for youth when compared to no social phobia or to other anxiety diagnoses (e.g., Stadelmann et al., 2017). The reasons for this are complex, and studies have shown that core elements of social phobia, such as fear of negative evaluation and avoidance of social situations, interfere with self‐concept development (e.g., van Tuijl et al. 2016). We also include anxiety and depressive symptom trajectories to be able to evaluate whether internalizing symptom improvement is associated with self‐concept improvement.

In terms of treatment‐related variables, we include treatment dropout, as this will give us an indication as to whether completing CBT plays a role in the long‐term self‐concept trajectory. We also include treatment format (i.e., ICBT vs. GCBT), to evaluate how different CBT formats may influence the self‐concept trajectory. Whereas most trials have found no outcome difference between ICBT and GCBT in terms of anxiety and depression outcomes (see Wergeland et al., 2021); these formats may affect the self‐concept differently. For example, the social peer support elements in GCBT may benefit youth's self‐concept. The self‐concept has been postulated to constitute cognitive schemata that are strongly influenced by feedback from the social environment (Orth & Robins, 2013).

We hypothesize that the self‐concept increases from pretreatment to posttreatment and continues to increase toward 1‐ and 4‐year follow‐up, in line with Taylor and Montgomery (2007). In terms of predictors, we expect that female sex, higher age, social phobia as a primary diagnosis, increase in anxiety and depressive symptoms, and dropout will be associated with a poorer self‐concept trajectory. In terms of treatment format, we explore this openly due to limited previous data.

## 1 METHODS

The data are from the Assessment and Treatment—Anxiety in Children and Adults study (ATACA; Wergeland et al., 2014), a randomized controlled trial examining the effectiveness of manualized CBT for youth with anxiety disorders. The ATACA trial was conducted across seven different out‐patient community clinics for youth in Norway.

### 1.1 Participants

The sample comprised 179 children and adolescents aged 8–15 years at pretreatment (*M* = 11.5 years, *SD* = 2.1; 53.6% girls; 46.4% boys). The family social class distribution was high (46.4%), medium (30.7%), and low (9.5%; Currie et al., 2008) with 13.4% not reported. Most (92.2%) reported European White ethnicity, whereas 1.7% reported Asian and 6.1% did not report ethnicity. The principal anxiety diagnosis was social phobia (46.9%), separation anxiety disorder (32.4%), or generalized anxiety disorder (20.7%).

### 1.2 Procedures

Participants were informed about the study during the clinics' routine intake procedures. Inclusion criteria were primary diagnoses of generalized anxiety disorder, separation anxiety disorder, or social phobia. Exclusion criteria were pervasive developmental disorder, psychotic disorder, and/or intellectual disability. Youths who used psychotropic medication were included if the dosage had been stable for at least 3 months before inclusion and kept constant during treatment (*n* = 11, 6.0%). Of 258 youths and parents invited for the initial evaluation, 221 accepted. Of those who accepted, 28 were found to not meet the inclusion criteria or meet at least one exclusion criterion, and 14 withdrew. Finally, 179 participants were randomized to treatment. Participants were assigned randomly in blocks of six from the younger (age 8–12 years) or the older (age 12–15) group to either ICBT (*n* = 77), GCBT (*n* = 67), or a 10‐week waitlist (*n* = 38). Participants in the waitlist condition were subsequently randomized to either ICBT or GCBT, so the final number of participants was *n* = 91 from ICBT and *n* = 88 from GCBT. Parents and all participants aged >12 years provided written informed consent/assent. Participants ages <12 years provided verbal assent. The study was approved by the Regional Committee for Medical and Health Research Ethics.

## 2 MEASURES

### 2.1 Anxiety Disorders Interview Schedule for Children and Parents

The Anxiety Disorders Interview Schedule (ADIS‐C/P; Silverman & Albano, 1996) was used to assess the inclusion diagnoses at pretreatment, posttreatment, 1‐year follow‐up, and 4‐year follow‐up. At 4‐year follow‐up, the adult version ADIS‐IV‐L (Brown et al., 1994) was used with participants above 18 years. Diagnoses and clinical severity ratings (CSR) were assigned based on combined parent‐ and youth‐report. The CSR scale ranges from 0 to 8, with a minimum score of 4 by at least one of the informants required for a diagnosis (Silverman & Albano, 1996). In cases of multiple anxiety diagnoses, the diagnosis with the highest CSR score was considered primary. The ADIS‐C/P has demonstrated excellent inter‐rater reliability, retest reliability, and concurrent validity (Silverman et al., 2001; Wood et al., 2002). The ADIS‐IV‐L has also demonstrated good to excellent reliability (Brown et al., 1994). In this study, the inter‐rater agreement (kappa) for the presence of an inclusion anxiety diagnosis ranged from 0.84 to 0.94 across the four measurement points.

### 2.2 Beck Self‐Concept Scale

The Self‐Concept Scale of the Beck Youth Inventories of Emotional and Social Impairment (SCS; Beck et al., 2001), youth‐report, was used to measure participants' self‐concept. The SCS comprises 20 items (e.g., *I feel good about myself*). Items are rated on a 4‐point scale (0 = never, 1 = sometimes, 2 = often, 3 = always). Lower values indicate lower self‐concept. The SCS measures self‐esteem and competency dimensions but is recommended used as a single component self‐concept measure (Steer et al., 2005). The scale has shown high internal consistency (Kornør & Johansen, 2016) and good test–retest reliability (Thastum et al., 2009). In our sample, internal consistency was *α* = 0.93.

### 2.3 Spence Children's Anxiety Scale

The Spence Children's Anxiety Scale (SCAS; Spence, 1998), youth‐report, was used to measure participants' anxiety symptoms. The SCAS comprises 38 items (e.g., *I am afraid of the dark*). Items are rated on a 4‐point scale (0 = never, 1 = sometimes, 2 = often, 3 = always). Higher values indicate more anxiety. SCAS has a 6‐month test–retest reliability of 60 (Spence et al., 2003; Spence, 1998). In our sample, internal consistency was *α* = 0.91.

### 2.4 Short Mood and Feelings Questionnaire

The Short Mood and Feelings Questionnaire (SMFQ; Angold & Costello, 1995), youth‐report, was used to measure participants' depressive symptoms. The SMFQ comprises 13 items (e.g., *I felt miserable or unhappy*). Items are rated on a 3‐point scale (0 = not true, 1 = sometimes true, 2 = true). Higher values indicate more depression. Two‐week test–retest reliability was 66 (Kuo et al., 2005). In our sample, internal consistency was *α* = 0.88.

All measures were administered at pre‐, post‐, 1‐year, and 4‐year posttreatment.

### 2.5 Treatment

The treatment followed a Norwegian version of the Friends for Life program, fourth edition (FRIENDS; Barrett & Ryan, 2004). FRIENDS is a manualized CBT program targeting cognitive, physiological, and behavioral factors that have been found to affect the development and maintenance of anxiety problems in children (Barrett & Ryan, 2004). The program was delivered as 10 weekly sessions with two booster sessions. The program is skills‐based, focusing on identifying and challenging negative thoughts, building problem‐solving skills, and teaching relaxation techniques. From Session 5, exposure techniques are also introduced. If participants missed sessions, they were offered catch‐up sessions. Participants absent from more than three consecutive sessions were considered dropouts.

### 2.6 Therapists and assessors

Seventeen therapists who were regular employees in the participating clinics delivered the treatments. Therapists had a mean of 10.8 years of clinical experience (*SD* = 6.3). Therapists participated in a 2‐day workshop on CBT and childhood anxiety disorders, a 2‐day workshop on the FRIENDS manual, and treated two pilot cases that were approved by supervisors before the study. During the study, all therapists participated in four additional 2‐day workshops on topics related to anxiety disorders. Supervision was conducted every 2–4 weeks by experienced CBT therapists licensed in the FRIENDS manual. Therapist adherence and competence were rated on a 7‐point scale (0 = none to 6 = thorough for adherence and 0 = poor to 6 = excellent for competence). All 17 therapists met the criterion of a minimum mean score above 3.0 for adherence and competence. Inter‐rater reliability was ICC = 0.80 for adherence and ICC = 0.68 for competence.

The assessors were experienced clinicians employed at the clinics. The assessors participated in workshops on CBT and anxiety disorders, and in a 2‐day ADIS‐C/P workshop with licensed ADIS‐C/P‐raters. The assessors were supervised two to four times per year and had biweekly phone contact with a supervisor to discuss administration and scoring during the study period. Masking of the assessors was not possible as they worked at the participating clinics.

### 2.7 Data analytic plan

We ran missing value analyses for all variables to assess the extent and patterns of missing data. All participants (pretreatment *N* = 179) were included in all analyses, whether they completed treatment (*n* = 155; 86.6%) or did not (*n* = 24; 13.4%). The number of participants with complete self‐concept data at each of the four assessment intervals was 163 at pretreatment (8.9% missing), 141 at posttreatment (21.2% missing), 133 at 1‐year follow‐up (25.7% missing), and 144 at 4‐year follow‐up (19.6% missing). Missing data for the variables age, sex, primary anxiety diagnosis, and anxiety/depressive symptoms measured at pretreatment did not exceed 7.7%. Missing value analyses were run at the measure level for all variables included in our main analyses. We ran Little's missing completely at random test to assess patterns of missing data (Little, 1988). The test indicated no systematic pattern in the missing data.

We ran descriptive analyses to assess violation of the normality assumption for the continuous variables included in our main analyses, as several of these analyses assume a normal distribution. Skewness and kurtosis values were well within the recommended range of ±3.29 (Kim, 2013) for all variables. The effect of outliers was assessed using 5% trimmed means. Means did not meaningfully differ when outliers were excluded, and they were thus kept in the further analyses.

We then ran a series of growth curve models using R version 4.1.2 for self‐concept across the four measurement points. We examined both within‐subject changes over time and between‐subject variation. In addition to evaluate changes in mean scores at the group level over time, we also wanted to investigate the individual growth trajectories (Gelman & Hill 2007). An advantage of this approach is that it enables the inclusion of participants with partial data (Heck & Thomas 2015). We investigated variances in both the intercept (i.e., the estimated initial status at pretreatment) and the slope (i.e., the estimated change over time). The trajectories for anxiety, depression, and self‐concept, respectively, were constructed by restructuring the separate timepoint measurements (pre‐, post‐, 1‐year, 4‐year post) into one continuous variable sorted by time (coded 0, 1, 2, 3).

We conducted the growth curve analyses in a series of stages. In a first model (Model 1), we included a random intercept and a fixed effect of time. In a second model (Model 2), we added fixed effects of the pretreatment variables age, sex, and social phobia as the primary diagnosis. In a third model (Model 3), we added the trajectories for anxiety symptoms (child‐rated SCAS). In a fourth model (Model 4), we added the trajectories for anxiety symptoms (child‐rated SCAS) and depressive symptoms (child‐rated SMFQ). In a fifth model (Model 5), we added treatment dropout. We included interaction terms with time for all predictors in all models. All regression analyses were conducted using full information maximum likelihood, to retain as much data as possible. The Akaike information criterion was used to indicate model fit.

We found that those randomized to ICBT had a higher self‐concept at pretreatment compared to those randomized to GCBT (*p* < 0.05, *d* = 0.32). Therefore, we controlled for the pretreatment self‐concept when treatment format (i.e., ICBT vs. GCBT) was added to Model 5. The pretreatment self‐concept was a significant predictor of the self‐concept trajectory (*β* = 0.60, 95% confidence interval [CI]: 0.50–0.66), whereas format was not (*β* = −1.33, 95% CI: −3.00 to 0.33). We decided to leave out treatment format as a predictor as potential effects would most likely be due to the pretreatment difference in self‐concept.

We computed effect sizes using the equation for Cohen's *d* ((*M*_1_ − *M*_2_) − pooled SD) and considered *d* = 0.80 large, *d* = 0.50 moderate, and *d* = 0.20 small (Cohen, 1992).

## 3 RESULTS

### 3.1 Correlations between variables

To contextualize the findings, we provide the frequencies for dichotomous variables, the means and standard deviations for continuous variables, and the correlations between all variables in Table 1.

**Table 1:** Study variables frequencies, means, and correlations.

	%/M (SD)	SoP	Sex	Age	Anxiety	Depr.	SC
SoP	46.9% yes	1	0.07	0.37**	−0.08	0.02	−0.19*
Sex	47.5% male	0.07	1	0.20**	0.21**	0.24**	−0.14
Age (years)	11.6 (2.1)	0.37**	0.20**	1	0.09	0.26**	−0.34**
Anxiety	36.3 (16.6)	−0.03	0.22**	0.09*	1	0.48**	−0.25**
Depr.	7.5 (5.5)	0.04	0.23**	0.18**	0.61**	1	−0.50**
SC	34.7 (11.3)	−0.13**	−0.13**	−0.29**	−0.40**	−0.60**	1

*Note*: For the correlations above the diagonal all variables were measured at pretreatment. For the correlations below the diagonal, anxiety, depression, and self‐concept were measured over time (pre‐, post‐, 1‐, and 4‐year posttreatment).

Abbreviations: M, mean; Depr., depression symptoms; SD, standard deviation; SCS, self‐concept; SoP, diagnosis of social phobia.

* Correlation is significant at the <0.05 level.

** Correlation is significant at the <0.001 level.

### 3.2 Self‐concept trajectory

**Figure 1:**
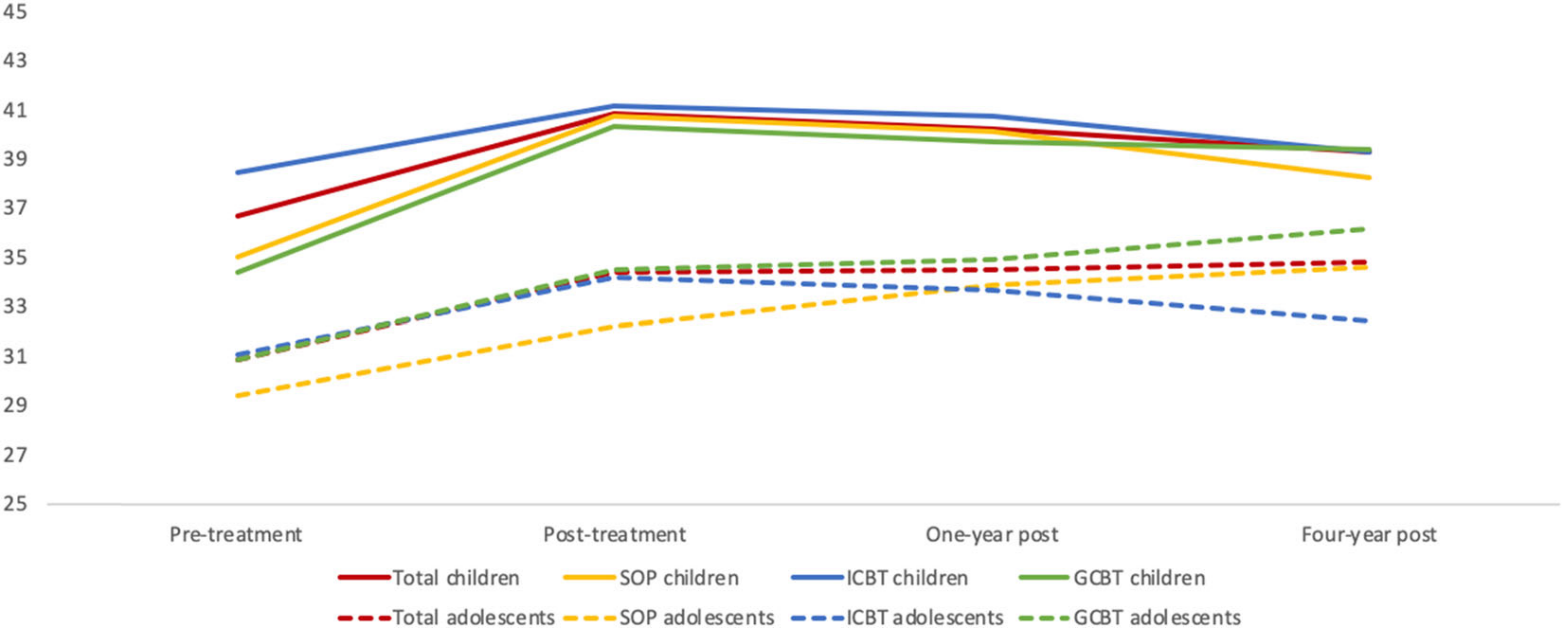
Self‐concept trajectories for the total sample, ICBT, GCBT, and diagnosis of social phobia by participant age note. Children >12 years; adolescents <12 years. GCBT, group cognitive behavioral therapy; ICBT, individual cognitive behavioral therapy.

See Figure 1 for the mean self‐concept scores at the various measurement points. We ran a growth curve model for the self‐concept trajectory showing a significant fixed effect of time. The main change in self‐concept occurred between pretreatment and posttreatment. The effect size differences in self‐concept change between pretreatment and posttreatment and pretreatment and long‐term follow‐up were small (*d* = 0.07 to 0.34). Thus, our data suggest a significant, albeit small, increase in participant self‐concept from pre‐ to post‐CBT that remained relatively stable 4‐year posttreatment.

### 3.3 Predictors of the self‐concept trajectory

We did the analyses predicting the self‐concept trajectory from pretreatment to 4‐year follow‐up as a series of growth curve models. After the pretreatment model (Model 1), the next model (Model 2) included pretreatment predictors, that is, youth age and sex, and primary diagnosis of social phobia. We then subsequently added the anxiety symptom trajectory (Model 3), depressive symptom trajectory (Model 4), and finally treatment dropout (Model 5) to examine if these variables enhanced model fit. See Table 2.

**Table 2:** Parameter estimates for the growth curve models for the self‐concept trajectory.

	Model 1		Model 2		Model 3		Model 4		Model 5	
	Est	95% CI	Est	95% CI	Est	95% CI	Est	95% CI	Est	95% CI
Fixed effects										
Intercept	**35.69**	34.02,37.36	**58.90**	46.21, 71.61	**61.77**	49.14, 74.43	**56.33**	1.87, 3.51	**46.20**	31.32, 61.15
Slope	**1.02**	0.43,1.62	−2.15	−6.89, 2.57	−2.75	−7.29, 1.74	−1.83	−0.89, 0.32	1.80	−3.83, 7.42
Age			**−1.78**	−2.60, −0.97	**−1.67**	−2.48, −0.87	**−1.26**	−0.42, −0.01	**−1.16**	−1.91, −0.42
Sex			−2.23	−5.37, 0.92	−1.01	−4.15, 2.14	−0.23	−3.11, 2.65	−0.23	−3.08, 2.62
Social phobia			0.55	−2.79, 3.88	1.05	−2.27, 4.36	1.37	−1.66, 4.39	1.40	−1.61, 4.68
Anxiety					**−0.19**	−0.29, −0.12	−0.07	−0.16, 0.01	−0.08	−0.16, 0.01
Depression							**−0.71**	−0.96, −0.46	**−0.70**	−0.95, −0.45
Dropout^a^									**4.73**	0.31, 9.16
Model fit										
AIC	4248.7		4232.0		4088.9		3948.8		3948.0	

*Note*: Significant estimates in bold.

Abbreviations: Anxiety, trajectory of the Spence Children's Anxiety Scale trajectory; C, child‐report; CI, confidence intervals (with lower and upper bound); Depression, trajectory of the Short Moods and Feelings Questionnaire; Est, estimates; Format, individual (coded 1) versus group (coded 0).

^a^ Dropouts coded 1, non‐dropouts coded 2.

In all models, age was a negative predictor of the self‐concept trajectory. There was no significant interaction effect between age and time (*p* = 0.103), which indicates that the self‐concept trajectory was similar for children and adolescents (see Figure 1). The anxiety symptom trajectory was a significant predictor for the self‐concept trajectory before the depressive symptom trajectory was included (Model 3). When the anxiety symptom and the depressive symptom trajectories were considered together, only the depressive symptom trajectory was a predictor (Models 4 and 5). In Model 5, treatment dropout was a significant predictor and enhanced model fit. Treatment dropout was associated with a self‐concept decrease over time. There was a significant interaction effect between time and dropout in Model 5 (95% CI: −3.6 to −0.2), indicating that participants who dropped out of CBT showed a reduction in self‐concept over time, whereas those who completed CBT showed an increase in self‐concept over time. Age and the depressive symptom trajectory remained significant predictors in Model 5.

Model 5, which included all predictors, showed the best model fit. However, the difference between the models with and without treatment dropout (Models 4 and 5) was not significant (*χ* ^2^ = 8.826, *p* = 0.065). We interpret the results to indicate that anxiety improvement predicted self‐concept increase, but only when considered separately from depressive symptom improvement. Improvement in depressive symptoms was associated with an increase in the self‐concept from pretreatment to 4‐year post‐CBT. Completing CBT also contributed positively to self‐concept improvement, but not above and beyond improvement in depressive symptoms. Across the models, self‐concept improvement was generally lower for older youth.

## 4 DISCUSSION

Among youth and adolescents with anxiety disorders, CBT was effective in increasing the self‐concept. and maintaining this gain up to 4‐year posttreatment. Our findings are in line with studies that have found increases in the self‐concept following CBT (Goldin et al., 2013; Shirk et al., 2003) and the FRIENDS program specifically (Barrett et al., 2003; Stallard et al., 2008). The most consistent predictor of the self‐concept trajectory was depressive symptom improvement. Whereas anxiety symptom improvement predicted self‐concept change, this effect disappeared when the depressive symptom trajectory was included. This indicates that the self‐concept change is mainly associated with the depressive symptom change.

Older age at pretreatment was associated with less self‐concept change. However, at the 4‐year follow‐up, the self‐concept scores had remained more or less constant since treatment completion, and there was no interaction effect between age and time. This indicates that CBT affects the self‐concept of both children and adolescents, and possibly prevents a self‐concept decrease typical for adolescence (Robins & Trzesniewski, 2005).

Sex was not a significant predictor of self‐concept in the current study. Maldonado et al. (2013) found that girls had lower self‐concept than boys, but this finding was not significant when controlling for the effect of anxiety or comorbidity. Thus, sex did not appear to be an important predictor of self‐concept in their clinical sample, which was in line with our finding. Overall, studies have found small to moderate sex differences in self‐concept (e.g., Bleidorn et al., 2016). The fact that potential sex effects are not large may help explain our null‐finding. In our sample, the fact that everyone had an anxiety disorder is likely to have outweighed potential small to moderate sex effects.

A primary diagnosis of social phobia did not predict the self‐concept trajectory. Previous studies have demonstrated poorer self‐concept in social phobia (e.g., de Jong et al., 2012; Maldonado et al., 2013). Our results imply that such an association is mainly cross‐sectional and may not apply over time, particularly not when considered alongside other variables.

Importantly, although the self‐concept trajectory and the depressive symptom trajectory demonstrated a significant overlap, this overlap was medium and similar to the overlap between anxiety and depressive symptoms. This indicates that self‐concept and depressive symptoms are related, but distinct phenomena, like depression and anxiety.

### 4.1 Strengths and limitations

The current study has a number of strengths. To our knowledge, we are the first to examine the self‐concept trajectory in child anxiety treatments in a community setting. Moreover, the study sample was large for a community setting, and longitudinal data up to 4‐year posttreatment is a strength. Most previous studies on self‐concept and anxiety are efficacy studies conducted with high experimental control in university settings. Therefore, our results may be more generalizable to CBT treatments offered in routine clinical care. Another strength is valid measurement assessments throughout the study, such as assessing symptoms and diagnoses at several measurement points. Diagnostic information was gathered through clinicians' evaluation of combined youth‐ and parent‐reports reducing the potential bias caused by differing perspectives, as studies have found small to moderate agreement between youth and parent ratings (De Los Reyes et al., 2009).

The study also has limitations. Our results cannot necessarily be generalized to other anxiety disorders than the inclusion diagnoses, other age groups, or other treatment programs than CBT or the FRIENDS program specifically. Another potential limitation is the use of a different self‐concept measure compared to most other studies. Others have primarily used the Rosenberg Self‐Esteem Scale (Rosenberg, 1965), whilst the present study used the Beck SCS (Beck et al., 2001). The most notable difference is the fact that they measure slightly different constructs, where the Beck Scale measures self‐concept, whereas the Rosenberg Scale measures self‐esteem. This could reduce comparability to other studies. Moreover, the treatment program was not designed to be a self‐concept treatment, which limits our ability to assess the potential of self‐concept treatment. Despite the randomized design, the pretreatment self‐concept was significantly different between ICBT and GCBT, which prevented us from examining the treatment format as a predictor as intended.

### 4.2 Implications and future directions

The current study has implications for practice. The fact that the self‐concept increase is lower with increasing age could mean that treatment targeting self‐concept is most effective as early intervention. It has also been suggested that the self‐concept is more malleable and easier to change through treatment in childhood and early adolescence, due to the relatively low stability of self‐concept in this period (Robins & Trzesniewski, 2005). This could mean that self‐concept interventions for at‐risk children should be implemented to a larger extent and that early detection of low self‐concept in children could be important to prevent further negative development.

The increased self‐concept following the FRIENDS program indicates that there is something to learn from the treatment delivered in the present study. Though it is uncertain why it works, some possibilities are its positive, resilience‐building orientation, trained therapists, or licensed supervisors, which perhaps should be implemented to a larger extent in community clinics. However, the small effect sizes and the lack of continued self‐concept increase after treatment completion indicate that the treatment could be more effective. The treatment could perhaps be improved by explicitly emphasizing self‐concept to a larger extent, increasing the number of sessions, or adding more follow‐up booster sessions to maintain and consolidate positive changes.

## 5 CONCLUSION

Our concluding summary from this study of the self‐concept in a community sample of children with anxiety disorders is as follows: Participants' self‐concept significantly increased from pretreatment to posttreatment and remained stable until long‐term follow‐up. The changes in self‐concept seem to be mostly associated with improvement in depressive symptoms. An important question for further research is how the self‐concept should be focused on in CBT as a transdiagnostic phenomenon, to further advance treatment options for youth with anxiety.

## CONFLICT OF INTEREST

The authors declare no conflict of interest.

### PEER REVIEW

The peer review history for this article is available at https://publons.com/publon/10.1002/jclp.23427

## ETHICS STATEMENT

The study was approved by the regional board for ethics in medical research. All parents consented on behalf of children aged <16 years. Participants aged >16 years consented.

## Data Availability

The data that support the findings of this study are available from the corresponding author upon reasonable request.
